# A Multi-Center, Cross-Sectional Study on the Burden of Infectious Keratitis in China

**DOI:** 10.1371/journal.pone.0113843

**Published:** 2014-12-01

**Authors:** Xiusheng Song, Lixin Xie, Xiaodong Tan, Zhichong Wang, Yanning Yang, Yuansheng Yuan, Yingping Deng, Shaoying Fu, Jianjiang Xu, Xuguang Sun, Xunlun Sheng, Qing Wang

**Affiliations:** 1 State Key Laboratory Cultivation Base, Shandong Provincial Key Laboratory of Ophthalmology, Shandong Eye Institute, Shandong Academy of Medical Sciences, Qingdao, Shandong Province, China; 2 Public Health College of Wuhan University, Wuhan, Hubei Province, China; 3 Zhongshan Ophthalmic Center of Sun Yat-sen University, Guangzhou, Guandong Province, China; 4 Department of Ophthalmology, Renmin Hospital of Wuhan University, Wuhan, Hubei Province, China; 5 Department of Ophthalmology, First Affiliated Hospital of Kunming Medical University, Kunming, Yunnan Province, China; 6 Eye Center, West China Hospital of Sichuan University, Chengdu, Sichuan Province, China; 7 Department of Ophthalmology, First clinical College of Harbin Medical University, Harbin, Heilongjiang Province, China; 8 Department of Ophthalmology, Eye and ENT Hospital of Fudan University, Shanghai, China; 9 Beijing Institute of Ophthalmology, Beijing, China; 10 The People's Hospital of Ningxia Hui Autonomous Region, Yinchuan, China; 11 Qinghai University Affiliated Hospital, Xining, China; Medical College of Soochow University, China

## Abstract

**Objective:**

To understand the prevalence and demographic characteristics of infectious keratitis and infectious corneal blindness.

**Methods:**

A multi-center, population-based cross-sectional study was conducted from January 1 to August 31, 2010. A total of 191,242 individuals of all age groups from 10 geographically representative provinces were sampled using stratified, multi-stage, random and systematic sampling procedures. A majority, 168,673 (88.2%), of those sampled participated in the study. The examination protocol included a structured interview, visual acuity testing, an external eye examination, and an anterior segment examination using a slit lamp. The causes and sequelae of corneal disease were identified using uniform customized protocols. Blindness in one eye caused by infectious keratitis was defined as infectious corneal blindness.

**Results:**

The prevalence of past and active infectious keratitis was 0.192% (95% confidence interval [CI], 0.171–0.213%), and the prevalence of viral, bacterial, and fungal keratitis was 0.11%, 0.075%, and 0.007%, respectively. There were 138 cases of infectious corneal blindness in at least one eye in the study population (prevalence of 0.082% [95%CI, 0.068%–0.095%]). Statistical analysis suggested that ocular trauma, alcoholic consumption, low socioeconomic levels, advanced age, and poor education were risk factors for infectious corneal blindness.

**Conclusions:**

Infectious keratitis is the leading cause of corneal blindness in China. Eye care strategies should focus on the prevention and rehabilitation of infectious corneal blindness.

## Introduction

The World Health Organization (WHO) reported in 2001 that corneal disease, as a major cause of blindness, ranks second only to cataracts worldwide [1]. In recent decades, following the implementation of various programs initiated by the WHO, rates of corneal diseases attributable to *Chlamydia trachomatis*, onchocerciasis, and leprosy have improved. Currently, infectious keratitis is mainly caused by viruses, fungi, bacteria, and *Acanthamoeba*. In developing countries, most patients with infectious keratitis have limited access to medical care. In addition, the lack of effective drugs, essential operating equipment, and well-trained medical care personnel, together with the lack of legislative guarantee and the shortage of corneal grafts, results in severe outcomes. It is estimated that trauma and corneal ulcers are responsible for 1.5–2.0 million new cases of corneal blindness every year, and this type of blindness has been recognized as a “silent epidemic” [2].

The First China National Sample Survey on Disability showed that cataracts and corneal diseases were the top two causes of blindness and that the prevalence of corneal blindness and low vision was 21/10,000. The survey also suggested that corneal blindness accounted for approximately 1/4 of blindness cases in China, with infectious keratitis being the major cause of corneal blindness [3]. Over the past two decades, following the rapid growth of the Chinese economy, great success in disease control has been achieved. The Chinese government and charity organizations launched many sight-restoring projects that focused on blinding eye diseases, but the prevention of corneal blindness has received less attention. To achieve the goal of the “VISION 2020-The Right to Sight” initiative, which was initiated by the WHO, the prevention of corneal blindness should receive more attention in China.

The prevention of infectious corneal blindness is important for the national control of corneal blindness. It is known that disease control strategies depend on epidemiological data. However, few national epidemiological surveys pertaining to infectious keratitis have been conducted in China. Funded by grants from the Chinese Academy of Engineering, the Shandong Eye Institute began an Epidemiological Study of Infectious Keratitis in China. This was the first national, multi-center, epidemiological survey of infectious keratitis. The aim of the study was to investigate the epidemiological data on infectious keratitis in China for the purpose of providing evidence to encourage the central and provincial governments to develop intervention strategies.

## Materials and Methods

### Study design

As there are currently no studies that report the prevalence of infectious corneal disease in China, a pre-survey was conducted in urban areas of Beijing and rural areas of Shandong and revealed an estimated prevalence of infectious corneal disease of approximately 0.4%. The sample size was calculated using the formula: n =  Z^2^p(1-p)/B^2^. For p = 0.004, B = 0.1p, and Z_0.05/2_ = 1.96, the sample size should be approximately 100000, and the response rate must be greater than 80%. Therefore, considering sample loss, the sample size must be at least 120,000. According to the results from the Fifth National Population Census in 2000, there is a total population of 0.44 billion in the 10 provinces (municipalities or autonomous regions), and the sampling fraction is approximately 3/10000.

**Figure 1:**
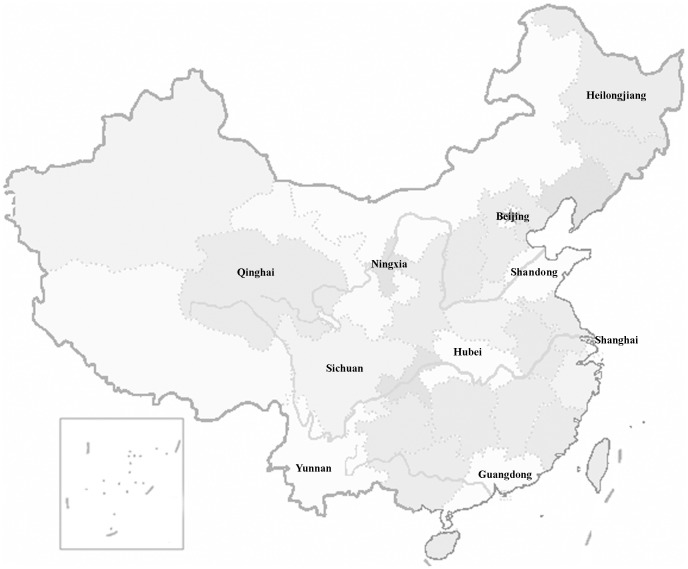
Distribution of the 10 provinces (municipalities and autonomous regions) in this study.

Multi-stage stratified cluster random sampling was adopted in the present study. In stage one, ten provinces in mainland China that represented different levels of socioeconomic development within the 31 provinces (municipalities or autonomous regions) were selected based on a list of provinces in each region and on computer-generated random numbers: 2 provinces (Shandong and Guangdong) and 2 municipalities (Beijing and Shanghai) were in the developed east coast region; 2 provinces (Hubei and Heilongjiang) were in the inland middle region; and 4 provinces (Sichuan, Yunnan, Qinghai, and Ningxia) were in the undeveloped west region (Fig. 1). The annual per capita consumption of urban residents in the 10 study areas ranged from ¥5,426 in Ningxia to ¥16,457 in Shanghai and from ¥1,404 in Ningxia to ¥7,516 in Shanghai among rural residents; the sampled study provinces were socioeconomically diverse [4]. In stage two, one district and one county were sampled within each province, and a subdistrict in the district and a township in the county were then selected. In stage three, communities or villages were randomly sampled from the subdistrict or townships. Following the methods in the published literature [5], the sampling frame was formed by geographically defined clusters based on community or village register data. Each cluster had a population of approximately 1,000 individuals (all ages). All clusters were then numbered and sorted, and simple random sampling was adopted to sample the clusters.

### Operating procedure

The field survey was carried out during the period from January 1 through August 31, 2010. The survey groups checked and confirmed the demographic data including names, genders, ages, and education levels registered by the communities or villages from house to house. Those who were not registered but who had lived in the clusters for more than 6 months were also included in the current survey.

The examinations were performed in special sites in the communities or villages, and the survey group was composed of pretrained doctors, nurses, and staff. All members of the survey group were trained prior to the investigation, including training on the standard survey procedure, visual examinations, the diagnostic standards for corneal disease, and a questionnaire. Guardians of the participants less than 18 years of age approved the survey on their behalf. Individuals who did not come to the examinations were either revisited and encouraged to participate in the study or they were examined at their homes using portable equipment, including a handheld slit lamp. The response rate was maintained at more than 80% in each cluster.

The field survey was conducted in two phases. In the first phase, presenting visual acuity was measured using the standard logarithmic visual acuity chart. For infants, colored toys were utilized. If there was no obvious abnormality in the eyes and the eyes could rotate with the movement of the toys, the baby was considered to not be blind. Those with visual disturbances, i.e., those whose eyes could not rotate with the movement of the toys, were diagnosed as having visual impairment. A slit lamp was used to examine the anterior segments of the eyes. Cases with corneal ulceration, infiltration, edema, scarring, opacity, vascularization, degeneration, pterygium, corneal transplantation, and foreign objects (arcus senilis was excluded) in the eyes or anophthalmos caused by corneal diseases were forwarded to the second-stage examinations.

In the second stage, the epidemiological questionnaire was completed, the anterior segments of the eyes were photographed with a digital camera or anterior segment camera, and the fundus was also examined. The epidemiological questionnaire contained queries regarding demographics, habits, and customs(smoking and alcoholicbeverage consumption), ocular trauma, and medical history, including ocular or systemic diseases. In the case of minors, the epidemiological questionnaires were completed by their guardians. When the corneal stromal infiltration (regardless of whether there was an epithelial defect) or the corneal ulcer was more than 1 mm^2^ (regardless of whether there was hypopyon), additional corneal smears and cultures were performed to make a definitive diagnosis [6].

The survey information and diagnostic results of each subject in the second phase were input into predesigned medical records. After the field investigation, each cooperation center checked the survey results to ensure the integrity and accuracy of the data and then logged in to the website created by the Shandong Eye Institute to upload files regarding the survey.

This study was approved by the ethics committees of the Shandong Eye Institute, the Zhongshan Ophthalmic Center of Sun Yat-sen University, the Renmin Hospital of Wuhan University, the First Affiliated Hospital of Kunming Medical College, the West China Eye Center of Sichuan University, and the First Clinical College of Harbin Medical University, and it complied with the Declaration of Helsinki. Informed consent was obtained from all participants following a detailed description of the purpose and potential benefits of the study prior to the examinations. Written consent was preferred, but if the participants were illiterate, verbal consent was recorded by our staff members. The ethics committee approved this consent procedure.

### Definition and criteria

#### Smoking and alcoholic beverage consumption

Smokers were defined as those who had smoked 100 cigarettes and now smoked either every day or some days. Alcoholic beverage consumption was defined as the consumption of more than one drink per day for women and more than two drinks per day for men. One drink was roughly equivalent to 300 ml beer, 150 ml wine, or 50 ml spirits.

#### Blindness and low vision

The WHO defines visual impairment as acuity less than 20/63, including blindness (visual acuity less than 20/400) and low vision (visual acuity worse than 20/63 but no less than 20/400). Considering that it was difficult to use the available methods and technologies to conduct the visual field tests due to the large number of participants in rural areas, the visual field test was not included in the present study.

#### Corneal blindness

Visual acuity less than 20/400 in one eye caused by corneal disease is defined as corneal blindness. A presumptive diagnosis was made by the professional doctors in the field based on medical history and examination results. The diagnosis and causes of corneal blindness were validated by specialists in the survey group based on the images and the survey data.

#### Diagnostic criteria of infectious keratitis

Active infectious keratopathy was diagnosed as follows: (1) bacterial, fungal, and *Acanthamoeba* keratitis were confirmed by etiological results, and (2) viral keratitis was diagnosed based on recurrent history, characteristic corneal lesions [7], and the clinical criteria for stromal herpetic keratitis [8].

#### Sequelae of past infectious keratitis

In cases with corneal opacity, scar, or anophthalmos, if the medical history or medical records demonstrated that the sequelae had been caused by bacterial, viral, fungal, *Acanthamoeba*, or other infectious keratitis, the diagnosis of sequelae of past infectious keratitis was made. The prevalence of corneal diseases (%) was calculated as follows: individuals with a history of corneal diseases plus new emerging cases/the subjects examined ×100%.

#### Statistical analysis

All data were entered into Excel (Microsoft Corporation; Redmond, WA, USA), and all statistical analyses were performed using the Statistical Package for the Social Sciences Version 17.0 (SPSS 17.0, SPSS Inc.; Chicago, IL, USA). Differences in prevalence were tested for statistical significance with the chi-square test, and those variables which were significant through univariate analysis were included into multivariate analysis. Multivariate analysis was conducted with binary logistic regression analysis. A P-value <0.05 was considered significant.

## Results

A total of 191,242 subjects were recruited from 10 provinces (municipalities or autonomous regions), and 168,673 individuals completed the investigation, corresponding to a response rate of 88.2%. The response rates ranged from 80.5% (Ningxia) to 96.8% (Hubei) in rural areas and from 80.0% (Yunnan) to 95.7% (Hubei) in urban areas. There was no significant difference in the characteristics between the individuals who completed and did not complete the survey based on the statistical analysis. Males and females accounted for 48.4% and 51.6% of the subjects examined, respectively; the ages ranged from 0 to 110 years, with a mean age of 40.5±20.5 years. The education levels were as follows: illiterate, 11.6%; primary school, 29.0%; middle school, 43.7%; and university and higher, 15.6%.

Based on the WHO definition of visual impairment, a total of 6,579 individuals were visually impaired; that is, they presented with a visual acuity of <20/63, with a visual impairment prevalence of 3.99% (95% CI: 3.89%–4.08%). Of the total cases with visual impairment, 525 had blindness (PVA <20/400), with a prevalence of 0.32% (95% CI: 0.29%–0.35%), and 6,054 had low vision (PVA ≥20/400, <20/63), with a prevalence of 3.67% (95% CI: 3.58%–3.76%).

There were 4,204 cases of corneal disease sequelae, with a prevalence of 2.49% (95% CI: 2.42%–2.57%). Corneal diseases were found more frequently among females (χ^2^ = 97.63, p<0.001), the rural population (χ^2^ = 337.87, p<0.001), and subjects with lower education levels (χ^2^ = 337.87, p<0.001). Subjects with advanced age were also found to have a higher prevalence of corneal disease (χ^2^ = 4046.85, p<0.001). The disease prevalence was higher in the eastern and western provinces than in the central regions (χ^2^ = 626.59, p<0.001) (Table 1). The major corneal diseases were pterygium (3,158 cases, 75.1%), infectious corneal disease (324 cases, 7.7%), and traumatic scarring (147 cases, 3.5%).

**Table 1:** Univariate analysis of the prevalence of corneal diseases.

	No. of participants	No. with corneal diseases (prevalence %, 95%CI)	χ^2^	P
**Gender**	168673		97.63	<0.001*
Male	81564	1720 (2.11, 2.01–2.21)		
Female	87109	2484 (2.86, 2.75–2.97)		
**Age**	168651 (No data for 22 person)		4046.99	<0.001*
0–14	20245	20 (0.10, 0.06–0.14)		
15–59	116245	1811 (1.56, 1.49–1.63)		
60 and over	32161	2373 (7.38, 7.09–7.66)		
**Education level**	168607 (No data for 66 person)		2456.85	<0.001*
Illiterate	19563	1388 (7.09, 6.73–7.45)		
Primary school	48989	1544 (3.16, 3.00–3.31)		
Middle school	73777	1094 (1.49, 1.40–1.57)		
University	13467	115 (0.85, 0.70–1.01)		
Higher	12811	63 (0.49, 0.37–0.61)		
**Rural vs. urban**	168673		337.87	<0.001*
Urban	74902	1282 (1.71, 1.62–1.80)		
Rural	93771	2922 (3.12, 3.00–3.23)		
**Socioeconomic level**	168673		626.59	<0.001*
Eastern area	82075	2417 (2.95, 2.83–3.06)		
Central area	39852	316 (0.79, 0.71–0.88)		
Western area	46746	1471 (3.15, 2.99–3.31)		

* statistically significant difference.

The prevalence of blindness in at least one eye caused by corneal disease was 0.23% (95% CI: 0.20%–0.25%). Infectious corneal disease ranked first among causes of corneal blindness, accounting for 36.4% of cases. Of the 379 cases of corneal blindness, 20 cases (5.3%) had no light perception, or anophthalmos.

**Figure 2:**
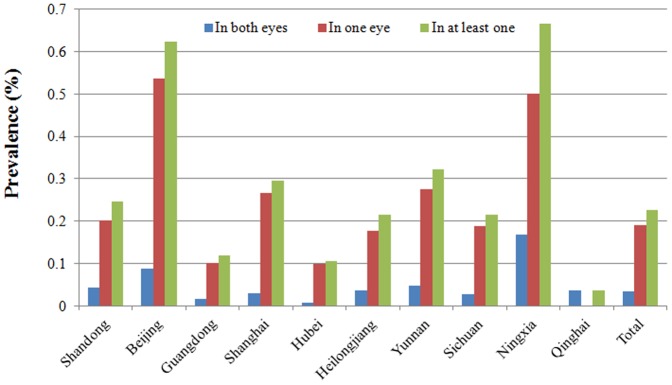
Geographic distribution of the prevalence of corneal blindness.

The prevalence of corneal blindness increased with age (χ^2^ = 739.7, p<0.001). Furthermore, corneal blindness had a female preponderance (χ^2^ = 5.25, p = 0.02) and was more common in the rural populations (χ^2^ = 60.74, p<0.001). The subjects with lower education levels had a higher prevalence of the condition (χ^2^ = 416.79, p<0.001) (Table 2). There was a significant difference in the prevalence of blindness among the eastern, central, and western areas (χ^2^ = 19.93, p<0.001), with the highest prevalence found in the western areas (Fig. 2).

**Table 2:** Demographic characteristics and the prevalence of corneal blindness.

Demographic distribution	No. of participants	Blindness in both eyes No. (%)	Blindness in one eye No. (%)	Total No. (%)
**Gender** a	168673			
Male	81564	22 (0.027)	139 (0.170)	161 (0.197)
Female	87109	35 (0.040)	183 (0.210)	218 (0.250)
**Age** b	168651 (No data for 22 persons)			
≤14	20245	0	0	0
15–59	116245	9 (0.008)	70 (0.060)	79 (0.068)
≥60	32161	48 (0.149)	252 (0.784)	300 (0.933)
**Education** c	168607 (No data for 66 persons)			
Illiterate	19563	29 (0.148)	132 (0.675)	161 (0.823)
Primary school	48989	22 (0.045)	117 (0.239)	139(0.284)
Middle school	73777	6 (0.008)	62 (0.084)	68(0.092)
University and higher	26278	0	11 (0.042)	11(0.042)
**Rural vs. urban** d	168673			
Urban	74902	12 (0.016)	81 (0.108)	93(0.124)
Rural	93771	45 (0.048)	241 (0.257)	286(0.305)
**Total**	168673	57 (0.034)	322 (0.191)	379(0.225)

a Comparisons between males and females. Blindness in both eyes, χ^2^ = 2.18, p = 0.14; blindness in one eye, χ^2^ = 3.48, p = 0.06; blindness in at least one eye(total), χ^2^ = 5.25, p = 0.02 (statistically significant difference).

b Comparisons between different ages. Blindness in both eyes, χ^2^ = 131.39, p<0.001 (statistically significant difference); blindness in one eye, χ^2^ = 608.80, p<0.001 (statistically significant difference); blindness in at least one eye(total), χ^2^ = 739.70, p <0.001 (statistically significant difference).

c Comparisons between different education levels. Blindness in both eyes, χ^2^ = 100.87, p<0.001 (statistically significant difference); blindness in one eye, χ^2^ = 321.00, p<0.001 (statistically significant difference); blindness in at least one eye(total), χ^2^ = 416.79, p≤0.001 (statistically significant difference).

d Comparisons between rural and urban areas. Blindness in both eyes, χ^2^ = 12.60, p<0.001 (statistically significant difference); blindness in one eye, χ^2^ = 48.43, p<0.001 (statistically significant difference); blindness in at least one eye(total), χ^2^ = 60.74, p<0.001 (statistically significant difference).

### Prevalence and characteristics of infectious keratitis and infectious corneal blindness

There were 324 subjects with sequelae of infectious keratitis and active ulcers, and the prevalences of infectious keratitis and herpes simplex keratitis were 0.19% (95% CI: 0.17%–0.21%) and 0.11%, respectively. Infectious keratitis was more commonly found in females (χ^2^ = 10.18, p<0.001), elderly subjects (15 years or older) (χ^2^ = 495.53, p<0.001), and those who had received less education (χ^2^ = 112.93, p<0.001). However, there were no significant differences found in the prevalence between urban and rural areas (χ^2^ = 0.43, p = 0.51) or between various economic development regions (χ^2^ = 5.50, p = 0.06) (Table 3). The prevalence of blindness in at least one eye caused by infectious keratitis was 0.08% (95% CI: 0.07%–0.10%).

**Table 3:** Univariate analysis of the prevalence of infectious corneal diseases.

	No. of participants		Number with infectious corneal diseases (prevalence %, 95%CI)	χ^2^ P
**Gender**		168673		10.18<0.001*
Male		81564	128 (0.16,0.13–0.18)	
Female		87109	196 (0.23,0.19–0.26)	
**Age**		168651 (No data for 22 person)		495.53<0.001*
0–14		20245	1 (0.005,0.00–0.01)	
15–59		116245	105 (0.09,0.07–0.11)	
60 and over		32161	218 (0.68,0.57–0.77)	
**Education**		168607 (No data for 66 person)		112.93<0.001*
Illiterate		19563	95 (0.49,0.39–0.58)	
Primary school		48989	101 (0.21,0.17–0.25)	
Middle school		73777	104 (0.14,0.11–0.17)	
University		13467	15 (0.11,0.06–0.17)	
Higher		12811	9 (0.07,0.02–0.12)	
**Rural vs. urban**		168673		0.43 0.51
Urban		74902	138 (0.18,0.15–0.21)	
Rural		93771	186(0.20,0.17–0.23)	
**Socioeconomic level**		168673		5.50 0.06
Eastern area		82075	175 (0.21,0.18–0.24)	
Central area		39852	60 (0.15,0.11–0.19)	
Western area		46746	89 (0.19,0.15–0.23)	

* statistically significant difference.

**Figure 3:**
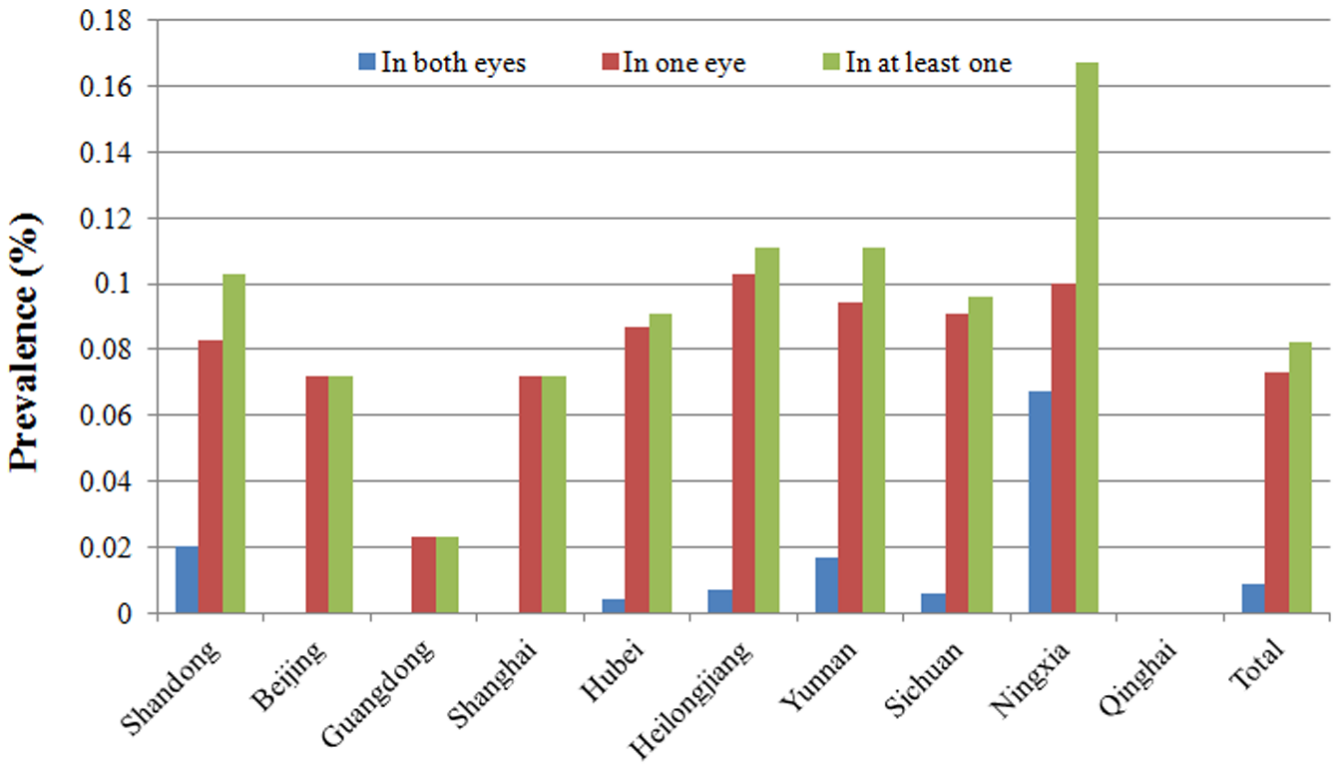
Geographic distribution of the prevalence of infectious corneal blindness.

A significant difference was observed in the prevalence of blindness caused by infectious keratitis among the various economic development regions (χ^2^ = 7.63, p = 0.02), and the lowest prevalence was found in the developed east coast region (Fig. 3).

There were studies showed that ocular trauma, alcoholic beverage consumption and smoking might be predisposing factors for corneal diseases [9]–[11]. In this study, univariate analysis showed that infectious keratitis caused a higher prevalence of blindness in rural areas (χ^2^ = 30.61, p<0.001) and among females (χ^2^ = 4.71, p = 0.03). The prevalence increased with age (χ^2^ = 260.59, p<0.001). Those who received less education had a higher prevalence (χ^2^ = 123.23, p<0.001). And higher prevalence was also found to be related to ocular trauma (χ^2^ = 4.89, p = 0.03), alcoholic beverage consumption (χ^2^ = 4.20, p = 0.04), and smoking(χ^2^ = 10.34, p = 0. 001). Furthermore, the logistic regression analysis suggested that ocular trauma, alcoholic beverage consumption, low economic levels, advanced age, and poor education were risk factors for infectious corneal blindness (Table 4). However, gender, hypertension, smoking, and rural vs. urban living were not related.

**Table 4:** Multivariate analysis of the risk factors for infectious corneal blindness.

Variables	B	S.E.	Wald	df	Sig.	Exp (B)	95% CI for Exp (B)	
							Lower	Upper
Education level	−0.188	0.077	6.015	1	0.014	0.828	0.713	0.963
Socioeconomic level	−0.450	0.154	8.600	1	0.003	0.637	0.472	0.861
Alcoholic beverage consumption	0.588	0.224	6.882	1	0.009	1.801	1.160	2.796
Ocular trauma	1.735	0.351	24.451	1	0.000	5.669	2.850	11.277
Age	0.051	0.004	150.933	1	0.000	1.052	1.044	1.061
Constant	−10.130	0.592	292.990	1	0.000	0.000		

### Estimates of the prevalence of visual impairment in the population

Based on the population of 1.3 billion revealed by the Fifth National Population Census in 2000 [12], it is estimated that approximately 51.87 million individuals are visually impaired in China, 4.16 million with blindness and 47.71 million with impaired vision. There were 32.37 million cases with sequelae and current corneal diseases, which had caused 2.99 million cases of blindness in at least one eye. Of the 2.47 million individuals with infectious keratitis (including sequelae and active keratopathy), 1.04 million were blind in at least one eye.

## Discussion

Corneal disease is a major cause of blindness worldwide. However, the prevalence and causes of corneal blindness vary in different countries, regions, and ethnic groups [1]. Two national sample surveys on disability conducted in China showed that corneal diseases ranked second (11.44%) and third (10.3%) in causes of visual impairment [3], [13]. According to the WHO definition, visual impairment is assessed by testing the eye with better visual acuity. However, corneal disease commonly presents in one eye and impairs vision unilaterally, leading to the underestimation of the actual burden of the condition. The present study is the first nationwide investigation conducted using a population-based, multi-center epidemiological survey in an attempt to understand the burden, causes, and population distribution characteristics of corneal blindness.

Pterygium, infectious keratitis, and traumatic corneal opacity are the most common corneal diseases reported in this study. Consistent with the population distribution of visual impairment reported by the WHO [14], [15], corneal diseases and corneal blindness were more prevalent among females, in rural areas, and among those who had received less education, and the prevalence increased with age. Daily jobs, living environments, and hygienic conditions, together with a lack of disease awareness and effective preventive measures, made these people prone to suffering from corneal lesions. Due to the lack of eye care, limited medical services, and poor economic conditions, the prevalence of corneal blindness is also high in these groups [16], [17]. To reduce the occurrence of corneal diseases and prevent corneal blindness, control strategies should focus on females in rural areas, less educated individuals, and the elderly. The western region had the highest prevalence of corneal blindness because this region is less economically developed.

Pterygium was the most common corneal disease (75.1%) and infectious keratitis was the most common cause of corneal blindness in this study. Up to one-third of the cases of corneal blindness were caused by infectious keratitis. It is estimated that 94.7% of the corneal blindness patients could have their sight restored through corneal transplantation. However, only a small number of hospitals in China have the necessary expertise, technology, and equipment for corneal transplantation. Furthermore, there is a lack of cornea donors in China. As a result, only approximately 5,000 corneal transplantation surgeries are performed annually [18]. Preventive measures against corneal diseases and early treatment for patients are needed to achieve the goal of the “VISION 2020: Right to Sight” initiative.

Infectious keratitis is the leading cause of corneal blindness in China. Traditionally, trachoma ranks first among infectious keratitis. In 1997, the WHO launched the program of “Global Elimination of Blinding Trachoma” (GET 2020) and developed a strategy known by the acronym “SAFE,” which stands for lid surgery (S), antibiotics to treat the infection (A), facial cleanliness (F), and environmental changes (E). Following the implementation of the strategy, the prevalence of trachoma was reduced [19], [20]. Bacterial, viral, and fungal corneal ulcers, as well as *Acanthamoeba* keratitis, are becoming the major sources of infectious keratitis that impairs vision [21], [22]. In the present study, the prevalence of infectious keratitis was 0.19% and the prevalence of herpes simplex keratitis was 0.11%. Herpes simplex keratitis was the leading infectious corneal disease that led to blindness in developed countries, with an annual incidence of herpes simplex keratitis ranging from 2.07/10000 to 3.15/10000 [7], [23], [24]. From 1950 through 1982, an epidemiological study of ocular herpes simplex virus infection was conducted in Rochester, Minnesota, USA. The survey showed that the prevalence of ocular herpes simplex virus infection in residents was 0.15% in 1980 [7]. A similar result was obtained in the present study, as the prevalence of herpes simplex keratitis was found to be 0.11%.

Infectious keratitis was mainly found in subjects living in poor economic conditions. However, the treatment cost of these conditions is very high. Moreover, there are often no effective drugs available to treat fungal corneal ulcers and *Acanthamoeba* keratitis in developing countries. In south India, approximately half of the cases of fungal keratitis resulted in blindness despite treatment [25]. With the emergence of resistant isolates and a decrease in effective drugs against bacteria, the rate of blindness associated with bacterial keratitis is also increasing. Because of the poor economic conditions and low health care awareness of females, rural residents, and less educated individuals, together with the lack of specialized doctors for corneal diseases in grassroots hospitals, the diagnosis and treatment of corneal diseases are very difficult. As a result, these residents have a higher prevalence of infectious corneal blindness.

In the present study, infectious keratitis was mainly found in less educated populations who mostly lived in rural areas and had little or no access to health care, and it mainly affected laborers. The disease not only impairs the body and mind but also imposes severe burdens on families and society. Corneal blindness is the final outcome of most infectious keratitis cases. It is of extreme urgency to emphasize the importance of prevention and control of infectious corneal disease and to reduce the number of cases of infectious corneal blindness. Prevention is the most cost-effective approach for reducing the incidence of infected corneal blindness in developing countries [26]. Prevention should focus on strengthening health education, realizing the risk factors and outcomes associated with infectious corneal disease, increasing awareness of eye health, and promoting occupational protection in workplaces. Because most cases of infectious keratitis are the result of corneal trauma, the use of 1% chloromycetin eye ointment for 3 successive days is recommended by the WHO to prevent the development of infectious keratitis [19], [26], [27].

The government should provide economic assistance for patients with infectious keratitis and increase medical insurance reimbursement. It was indicated that infectious keratitis was mainly found in populations with low socioeconomic status [13], [25], [28]. The high cost of corneal transplantation surgery, together with the direct and indirect economic loss caused by the disease, imposes a heavy burden on patients, their families, and society. Increasing the reimbursement percentage and economic assistance to patients would help reduce the incidence of infectious corneal blindness.
